# An analytical framework for examining the ethical dimensions of innovative humanitarian finance

**DOI:** 10.1186/s41018-026-00195-2

**Published:** 2026-04-17

**Authors:** Laila Zulkaphil

**Affiliations:** Humanitarian and Conflict Response Institute, University of Manchester, Manchester, United Kingdom

**Keywords:** Innovative humanitarian finance, Impact bonds, Blended finance, Ethics of humanitarian action, Market ethics, Humanitarian principles, Moral philosophy

## Abstract

Despite the increasing exploration of innovative financing mechanisms to address the widening humanitarian funding gap, scholarly engagement with innovative humanitarian finance remains remarkably scarce. Many of these mechanisms adopt blended finance models, in which public or philanthropic funds are used to de-risk and attract private investment into fragile contexts. While such arrangements raise fundamental questions about the compatibility of profit motives with humanitarian values, their ethical implications are not well understood. This article addresses these gaps by introducing a novel analytical framework for examining the ethics of using market-based financing mechanisms for humanitarian action. The framework integrates three interdisciplinary ethical perspectives – market ethics, humanitarian principles, and normative moral philosophy – to form a layered and comprehensive approach. The article demonstrates the application of the framework through the case of impact bonds – an outcome-based financing mechanism in which investors provide upfront capital and are repaid with interest if projects achieve their intended outcomes.

## Introduction

Global humanitarian funding has failed to keep pace with escalating needs driven by violent conflicts, climate and geological hazards, and food insecurity (Rieger et al., 2024). In 2023, for instance, only 45% of the humanitarian funding appeal coordinated by the United Nations (UN) was met, leaving a $30 billion shortfall – the largest recorded funding gap to date (OCHA 2025). To address the widening funding gap, the UN Secretary General commissioned a High‑Level Panel on Humanitarian Financing in 2016, which recommended innovative financing mechanisms such as impact bonds, micro levies, and Islamic finance (High-Level Panel on Humanitarian Financing 2016). The same year, the Grand Bargain – an agreement among humanitarian donors and agencies first adopted at the World Humanitarian Summit in Istanbul – called for more multi-year and flexible funding, while the Grand Bargain 3.0, revised in 2023, committed to exploring innovative financing approaches (Grand Bargain 2024). Recent government aid cuts, including the abrupt dismantling of the United States Agency for International Development (USAID), have reinforced the view that innovative approaches to financing humanitarian response, particularly through capital markets, are not merely an option but the only viable path forward (Meldrum 2025).

In response to these challenges, a number of humanitarian actors have piloted new financing mechanisms and approaches such as impact bonds (International Committee of the Red Cross, Near East Foundation), disaster insurance schemes (International Federation of Red Cross and Red Crescent Societies), refugee investment facilities (Danish Refugee Council), and technical advisory models (International Rescue Committee) (Farber et al. 2024). Despite its growing significance, scholarship on innovative humanitarian finance remains scarce. Existing sources are predominantly descriptive grey literature (Willitts-King, Bryant and Adamczyk, 2019; Farber et al. 2024; ODI 2024) that outlines emerging financing models without scrutinizing their impact or ethical dimensions. Academic literature that critically engages with innovative financing models is strikingly limited (namely, Ahmed 2021), underscoring the need for further inquiry.

This article contributes to addressing this knowledge gap by proposing a novel analytical framework that can be used by researchers and practitioners alike to examine the ethical dimensions of using innovative financing mechanisms for humanitarian action. Market-based financing approaches such as impact investing, disaster insurance, and other forms of blended finance often involve financial returns for the private sector, blurring the line between giving and profiting (Barnett 2023). Such models leverage public or philanthropic funds to de-risk and attract private capital, raising ethical concerns over the use of scarce humanitarian resources to subsidize commercial returns and the compatibility of profit motives with humanitarian values. Furthermore, the broader marketization literature indicates that the insertion of profit motives into an area previously governed by non-market norms such as public service delivery introduces a range of ethical risks, including perverse incentives that lead to ‘gaming’ the system to generate profit and subordination of service users’ needs to commercial interests (Joy and Shields 2018; Carter 2021; Krachler et al. 2022). Therefore, scholarship on innovative humanitarian finance must not be limited to performance evaluations – whether new financing mechanisms work in a technical or financial sense – but must also engage in ethical inquiry into how they work, for whom they work, and at what cost.

The proposed analytical framework adopts an interdisciplinary approach that integrates market ethics, humanitarian principles, and moral philosophical traditions, using a scaffolding method in which each ethical perspective addresses the limitations of the previous one. The adoption of innovative financing mechanisms in the humanitarian sector creates a new market, in which humanitarian action is packaged, priced, and sold to market actors as new investment or insurance products. Accordingly, I take market ethics as the starting point of the framework. In particular, the first building block of the framework is Debra Satz’s (2010) theory of noxious markets, which outlines four parameters that render a market ethically problematic. Satz contends that markets become unethical not because certain goods are inherently unsuitable for commercial exchange, but due to the structural conditions of market participants and the harmful consequences such markets produce. However, this dismissal of intrinsic value sits uneasily with humanitarian ethics, which are grounded in the belief that human life and dignity have intrinsic worth. To address this shortcoming, I introduce a fifth parameter inspired by Michael Sandel’s (2012) theory of the corrosive effect of markets, which highlights how marketization can erode the intrinsic value of certain goods.

Market ethics alone cannot determine whether financing mechanisms are compatible with the distinctive moral commitments of humanitarian action. Therefore, I turn next to the core humanitarian principles – humanity, neutrality, impartiality, and independence – by taking them as a proxy for humanitarian ethics to assess whether financing mechanisms can be considered humanitarian in a normative sense. Yet, because these principles are sometimes contested or come into tension with one another, I further extend the framework by drawing on broader traditions of moral philosophy – virtue, deontological, and consequentialist ethics – which provide a universal ethical perspective to address the gaps left by both market ethics and humanitarian principles. Together, these perspectives create a layered analytical framework that moves from the specific (market transactions) to the sectoral (humanitarian ethics) to the universal (moral philosophy), enabling a comprehensive and nuanced ethical examination of innovative humanitarian finance. Sect. " Theoretical underpinnings of the analytical framework" of the article discusses each ethical perspective in depth.

Sect. " Presenting the analytical framework" discusses how these ethical perspectives are integrated to generate the five parameters of the analytical framework: (1) understanding of the innovative financing mechanism by humanitarian actors, (2) agency of humanitarian actors involved, (3) the extent to which the mechanism aligns with the needs of affected populations, (4) costs and benefits of the mechanism, and (5) its implication for the intrinsic value of humanitarian action and the moral character of aid workers. I demonstrate the application of the analytical framework through a worked example of impact bonds – an outcome-based financing mechanism in which investors provide upfront capital and outcome funders repay with interest if results are achieved. Sect. " Conclusion" concludes the article by discussing the contributions, practical implications, and limitations of the framework.

## Theoretical underpinnings of the analytical framework

This section introduces the framework’s conceptual foundations – market ethics, humanitarian principles, and moral philosophy – which together support an interdisciplinary ethical assessment of innovative humanitarian finance. While alternative perspectives such as empirical ethics or post‑colonial ethics offer valuable insights, they are not adopted here as primary analytical lenses because the aim of the paper is to evaluate the ethicality of innovative financing mechanisms, which requires a normative rather than descriptive or practice‑based orientation. Moreover, concerns central to these approaches – such as situated ethical reasoning and structural power asymmetries – are already addressed within the framework through virtue‑ethical notions of practical wisdom and Satz’s focus on vulnerability, agency, and exploitation in noxious markets.

### Market ethics

Critiques of market functioning often adopt an economic lens narrowly focused on efficiency. For instance, Heath (2023) argues that markets become unethical when actors exploit market failures such as monopolies or negative externalities because doing so undermines efficiency, i.e. the moral foundation of markets. However, such accounts treat markets simply as production and distribution of goods and do not acknowledge that markets are also social institutions shaped by relationships, power dynamics, and values. Classical political economists, including Adam Smith, David Ricardo, and Karl Marx, emphasized how markets are socially embedded and how they influence human motivations, capacities, and relationships (Satz 2010). Following this tradition, I draw on two contemporary theories of market ethics that foreground the social relations underpinning market exchange. Satz’s (2010) theory of noxious markets argues that markets become unethical when they arise from or reinforce exploitative or unequal social relationships, while Sandel’s (2012) theory of the corrosive effect of markets highlights how commodification can corrupt the intrinsic value of certain goods and reshape people’s attitudes and behaviors. These theories are discussed in more detail below.

#### Theory of noxious markets

In her book, *Why Some Things Should Not Be for Sale: The Moral Limits of Markets*, Satz (2010) presents four parameters that make a market noxious or unethical: (1) underlying vulnerability and (2) weak agency of some market participants; and (3) individual harm and (4) societal harm produced by the market. Satz argues that scoring high along even one parameter would push a market into the noxious category. Following Morley’s (2021) article, which uses Satz’s theory to evaluate the ethical status of social impact bonds (where governments commission the former to finance public services), I adapt the original theory for the humanitarian context. Satz’s theory offers a robust foundation for assessing innovative humanitarian finance through the lens of market ethics because it moves beyond simplistic condemnations of marketization and instead provides a nuanced framework for evaluating the ethical dimensions of market structures. Satz is primarily concerned with distribution of power and agency within markets, which is crucial in humanitarian contexts, where affected populations and frontline actors may have limited control over the design and delivery of aid when market actors enter with greater resources and leverage. Her framework prompts questions such as who benefits from innovative humanitarian finance, who bears the risks, and to what extent commercial interests constrain the agency of humanitarian actors or undermine the needs of affected populations.

The first two parameters of Satz’s theory look at the sources of a market or ‘the underlying conditions of market agents’ (Satz 2010, p. 9). In particular, Satz argues that noxious markets often arise from underlying vulnerabilities, such as when poverty and desperation force someone to sell a kidney. When market participants come with significantly varying resources and capacities to understand the terms of their transaction, the weaker party is at risk of being exploited. Furthermore, a person entering into a contract from a position of extreme vulnerability is likely to accept almost any terms offered. Another characteristic of a noxious market is weak agency, which Satz defines as ‘very weak or highly asymmetric knowledge and agency on the part of market participants’ (Satz 2010, p. 96). In this category, Satz includes markets in which some parties have poor information about the goods they are exchanging. For example, in a surrogacy contract, a woman who has not been pregnant before cannot really know the consequences of giving up the child she bears. Satz also includes in this category markets in which some parties lack agency and cannot act independently, such as a market for child labor in which parents transact on behalf of children whose labor is traded.

The third and fourth parameters of Satz’s theory look at the consequences of a market. Specifically, she argues that harmful outcomes for individuals or society make a market noxious. Some noxious markets produce harmful outcomes either for the market participants themselves or for third parties, leaving the affected individuals destitute or undermining their basic needs and interests (Satz 2010). As an example, Satz refers to a grain market in which some people starve because they cannot afford the grain price determined through supply and demand. Noxious markets can produce harmful outcomes not only for individuals, but also for society: they undermine the social framework needed for people to interact as equals, supporting relations of subordination or unaccountable power. For example, Satz argues that child labor is harmful for society because it drives down adult wages, which, in turn, pushes more parents to send their children to work. Similarly, bonded labor produces harmful outcomes not only for the participants but also for society as it subjects people to humiliating subordination and undermines their capabilities to exercise their rights and participate in society (Satz 2010).

#### The corrosive effect of markets

Although Satz’s theory of noxious markets provides a useful framework for assessing whether innovative humanitarian financing mechanisms constitute an ethical market, it has limitations. Satz (2010, p. 117) explicitly rejects the ‘essentialist’ view that some goods have an intrinsic value that makes their commodification wrong – that is, their very nature or ‘essence’ dictates that they should not be bought and sold. For example, Satz argues that a market for women’s reproductive labor through contract pregnancy is unethical, not because there is something intrinsic about reproductive labor that makes its commodification demeaning, but because it places women’s bodies under the control of others and perpetuates gender inequality. Satz’s exclusive focus on outcomes and structural conditions, coupled with her rejection of the view that certain goods possess intrinsic value making their commodification wrong, is at odds with humanitarian ethics. The moral foundation and ethical goal of humanitarian action is to protect and respect human life because it is a fundamental good in itself, not because it is a means to some other end (Slim 2015a, p. 56). Therefore, an ethical examination of innovative humanitarian finance would be incomplete without taking into account this intrinsic value of humanitarian action.

For this reason, I adapt and expand Satz’s theory by adding a fifth parameter based on Sandel’s (2012) theory of the corrosive effect of markets. In his book *What Money Can't Buy: The Moral Limits of Markets*, Sandel (2012) argues that certain things in life have an intrinsic value, which can become corrupted or degraded when those goods are turned into commodities. Commodifying these goods can alter their meaning, change people’s attitudes toward the goods being exchanged, and crowd out moral values and norms (Sandel 2012). The corrosive effect of markets is not Sandel’s original idea. Hirsch (1976, p. 87) coined the term ‘the commercialization effect,’ which refers to ‘the effect on the characteristics of a product or activity of supplying it exclusively or predominantly on commercial terms rather than on some other basis – such as informal exchange, mutual obligation, or altruism.’ However, Sandel expands and illustrates this concept with examples of how markets leave their mark by altering the meaning and characteristics of goods being exchanged. For example, when a financial incentive is offered for giving blood, it is no longer seen as a noble act to help save lives. Instead, blood becomes just another commodity that is bought and sold on the market, resulting in the overall reduction of blood supply (Titmuss 1970). In other words, market mechanisms drive out the moral values that motivated people to donate blood in the first place (Sandel 2012). Therefore, by highlighting how markets can corrode intrinsic values of goods and reshape moral norms, Sandel’s account directly addresses the gap left by Satz’s framework.

### Humanitarian principles

Building on market ethics, I incorporate humanitarian principles as a sector-specific lens for assessing whether market-based financing mechanisms are compatible with principled (and ethical) humanitarian action. In some interpretations, the four foundational humanitarian principles – humanity, impartiality, independence, and neutrality – are constitutive of humanitarianism itself, and actors or actions that do not subscribe to these principles arguably fall outside the bounds of what can be legitimately labelled ‘humanitarian’ (Bradley 2023). Others, however, maintain that the principles were not meant to be precise prescriptions of practice but act as markers of value and guidance in imperfect situations (Slim 2015b). Although the continued relevance of the principles has been questioned in the face of the changing nature of conflicts and humanitarian action, they still serve as a useful ethical guide and help ensure access to affected populations (Weiss 2013; Carbonnier 2015; Gordon and Donini 2015; Slim 2015a). At the core of humanitarian ethics is a conviction that ‘every human life is good and that it is right to protect and save people’s lives whenever and wherever you can’ (Slim 2015a, p. 25). This conviction is expressed in the principles of humanity and impartiality and operationalized through the principles of independence and neutrality (Pictet 1979). Even if one rejects the principles because of changing times or other competing moral imperatives, their ethical underpinnings – particularly the inherent worth and dignity of every human being – cannot be dismissed. Therefore, the principles remain central to humanitarian ethics and provide a normative benchmark for determining whether new financing mechanisms preserve or undermine the ethical commitments that underpin humanitarian action.

#### Humanity

Humanity is the primary humanitarian principle, from which all other principles flow (Pictet 1979). Jean Pictet, the architect of the fundamental principles of the Red Cross and Red Crescent Movement, defined humanity as follows: ‘to prevent and alleviate human suffering wherever it may be found… to protect life and health and to ensure respect for the human being’ (Pictet 1979, p. 5). It is the least controversial of the four principles, but its broad acceptance also means that it is often unclear how to operationalize it in practice (Fast 2015). Therefore, I discuss below the principle of humanity and its related concepts of dignity, solidarity, and accountability.

The last part of Pictet’s definition of humanity, ‘respect for the human being,’ is significant as it implies that humanity is not simply about delivering humanitarian assistance, but is also about providing assistance in a respectful and dignified manner (Fast 2015). Pictet criticizes hospitals for treating sicknesses instead of individuals, regarding individuals simply as ‘numbers,’ and neglecting the human relations between those giving and receiving treatment (Pictet 1979, p. 25). Delivering humanitarian assistance in a manner that respects the dignity of individuals means humanitarians ought to demonstrate their closeness, presence, and solidarity with affected populations (Fast 2015). Hopgood (2008) argues that humanitarian service delivery is not just about efficiency, and that the deliverer is required to authentically feel somehow emotionally engaged because what they do is an ethical social practice. Otherwise, Wal-Mart, which has provided emergency response in a number of instances, becomes a humanitarian actor similar to World Vision (Hopgood 2008). Therefore, applying the principle of humanity as an ethical lens helps examine whether innovative financing mechanisms, by privileging market notions of efficiency, undermine the dignity of aid recipients and affect the human relations between those delivering and receiving aid.

Furthermore, operationalizing the principle of humanity entails valuing the local context, culture, and knowledge as well as giving and seeking information from local populations (Fast 2015). This concept is commonly known as ‘accountability to affected populations,’ which prescribes that humanitarian actors design their actions in consultation with affected populations as people can and should be able to articulate their own needs in crises (CHS Alliance 2021). However, innovative financing mechanisms bring together diverse actors such as investors, insurers, donors, and intermediaries, along with implementing agencies. As decisions are taken further from the effect, target populations risk being excluded from critical decisions that affect their lives. Humanitarian actors may identify and advocate for the needs of communities, but potential power asymmetries raise questions as to who ultimately controls program decisions. Therefore, the concept of accountability to affected populations provides an ethical benchmark for assessing whether innovative financing mechanisms prioritize the needs of communities over the commercial interests of market actors.

#### Independence

The principle of independence means operational autonomy, whereby humanitarians make their own assessments and decisions without influence from external actors (Slim 2015b). Pictet imagined that such external actors trying to influence humanitarians would be governments and intergovernmental organizations (Pictet 1979). Indeed, states have directly and indirectly constrained and steered humanitarian actors in ways that compromise humanitarian principles (Barnett 2005). Financing a humanitarian action through market-based mechanisms potentially introduces a new type of external influence, this time by the private sector.

According to Scott-Smith (2016), humanitarian principles distinguish the value-driven sphere of humanitarianism from the interest-driven spheres of politics and profit, and carve out an autonomous space that allow aid workers to prioritize human suffering over other interests. He argues that the humanitarian innovation movement in partnership with the private sector is eroding this independence of humanitarian actors. In a more optimistic tone, Barnett (2009) trusts that humanitarian actors’ ethical agency and capacity for critical inquiry will safeguard against possible moral complicity. Accordingly, the principle of independence serves as an ethical lens for assessing whether innovative humanitarian finance – by creating resource dependence on the private sector and injecting commercial interests into humanitarian action – erodes the independence of humanitarian actors or whether humanitarians are able to safeguard their independence and walk away from pressure if needed.

#### Neutrality

As per the principle of neutrality, humanitarian agencies must ‘not take sides in hostilities or engage at any time in controversies of a political, racial, religious or ideological nature’ (Pictet 1979, p. 6). Unlike the principle of humanity, neutrality does not in itself have a moral value. Instead, it is an operational principle, where the rationale for not taking sides in a conflict or engaging in any political controversies is for the humanitarian actor to enjoy the confidence of, or acceptance by, all the parties so that it can assist the affected populations (Pictet 1979).

No other humanitarian principle has caused as much debate and controversy as neutrality, to the point that it became a ‘dirty word’ – remaining silent in the face of atrocities is seen as complicity in crime (Fox 2001, p. 277). The International Committee of the Red Cross (ICRC) came under harsh criticism for failing to denounce the Holocaust during World War II, interpreting neutrality as silence. Since then, the organization has redefined neutrality as discretion rather than silence – remaining quiet in public while engaging in behind‑the‑scenes diplomacy on human rights abuses (Forsythe 2024). Yet this reinterpretation did not shield it from further criticism. The ICRC again faced condemnation for refusing to denounce government abuses during the Nigeria-Biafra conflict in 1968–70 and the Ethiopian famine in 1983–85 (Barnett and Weiss 2011; Krause 2014). The ICRC’s public silence and agreement to operate within the given constraints was seen as violating neutrality by abetting these governments’ war strategies. Disagreement over the interpretation of neutrality has, in turn, given rise to a new generation of humanitarian actors such as Médecins Sans Frontières, Oxfam, and ActionAid that embrace a more political approach and commit to bearing witness and speaking out (Fox 2001; Krause 2014). Others, however, with the ICRC at the forefront, emphasize the value of neutrality in enabling humanitarian access in hard‑to‑reach areas and continue to defend it as a core principle of humanitarian action (ICRC 2025).

These controversies have predominantly been related to military neutrality that entails refraining from taking sides in hostilities. Another meaning, related to ideological neutrality, stipulates that a humanitarian agency must not endorse any doctrine except its own as doing so would compromise its universal character (Pictet 1979). The analytical framework primarily engages with this meaning of neutrality since financing humanitarian action through market-based mechanisms has an ideological dimension. Such mechanisms are an extension of neoliberalism – a political and economic ideology that seeks the retreat of the state and the expansion of the market in its place by inserting or installing markets as the underlying mechanism for organizing society (Harvey 2005). Therefore, the drive for innovative finance risks undermining the ideologically neutral and universal character of humanitarian actors.

#### Impartiality

The principle of impartiality dictates that a humanitarian agency ‘makes no discrimination as to nationality, race, religious beliefs, class or political opinions’ and ‘endeavors only to relieve suffering, giving priority to the most urgent cases of distress’ (Pictet 1979, p. 5). While the principle of humanity asserts the intrinsic value of a human, the principle of impartiality ‘makes plain that this value belongs not just to some human beings but to every human person’ (Slim 2015b, p. 17). In other words, a humanitarian agency should not treat individuals differently because they belong to a certain category and must deliver assistance based on need only.

Innovative finance, particularly outcome-based financing mechanisms such as impact bonds in which payment is tied to the achievement of results, opens the door to a new type of discrimination – selection of aid recipients based on their ability or chances to succeed. However, prioritizing aid recipients based on their likelihood of success is an ethically grey area, as some might contend that it does not undermine the principle of impartiality by invoking parallels to medical triage, where resources are allocated according to individuals’ chances of survival rather than the severity of their needs alone (Pictet 1979). When allocating limited resources, should humanitarians prioritize those in the most dire circumstances, despite low odds of success, or those with better prospects for recovery, maximizing tangible impact? Pictet defers this moral dilemma to aid workers, trusting their discretion to weigh competing imperatives. Furthermore, Rubenstein (2015, p. 145) rejects the literal interpretation of the principle of impartiality as ‘aid based on need alone’ on the premise that ‘it is a poor instantiation of egalitarian, democratic, justice-based, and (perhaps surprisingly) humanitarian norms.’

These debates reveal a broader challenge – while humanitarian principles provide essential guidance, they sometimes fall short in addressing ethically ambiguous situations or reconciling competing priorities. Moral philosophy can fill this gap by providing specific frameworks to navigate ethical uncertainties, such as weighing consequences (consequentialism), adhering to duty (deontology), or prioritizing character-driven choices (virtue ethics). Therefore, I turn to moral philosophy as the next layer of the analytical framework.

### Moral philosophy

Ethics, which comes from the Greek word *ethos* meaning character, makes judgments about the character of humans and human actions and seeks to establish what is good and bad in human life and society (Slim 2015a). The branch of philosophy that studies ethics is called moral philosophy, and its sub-branch – normative moral philosophy – seeks to answer how people should behave and what makes an action morally right or wrong (Law 2007). The three main traditions of normative moral philosophy are virtue, deontological, and consequentialist ethics. When determining the morality of actions, these traditions respectively look at the character of people who undertake the actions, the actions themselves, and the consequences of actions. This section introduces these philosophical traditions and discusses their relevance to humanitarianism broadly and to innovative humanitarian finance specifically.

#### Virtue ethics

Founded by ancient Greek philosopher Aristotle, virtue ethics are concerned with moral character and view actions as expressions of virtue. Virtue ethics judge an action to be right if it is in character for a virtuous person – someone who has morally good traits of character (Law 2007; Bruni and Sugden 2013). Therefore, a virtuous action is an action that a virtuous person would undertake. According to Aristotle, virtue is the ‘golden mean’ between two extremes, which can be understood as a middle ground between two contrary vices – one of excess and the other of deficiency. For example, courage is the mean between over-confidence and cowardice, and generosity is the mean between miserliness and prodigality (Aristotle 2014). Moreover, virtues are of two kinds: intellectual virtues include understanding, judgment, and wisdom, whereas moral virtues include liberty and temperance, for instance (Aristotle 2014).

Virtue ethics do not provide rules or guidelines for determining what constitutes an ethical action. Instead, a virtuous person is guided by *phronesis* or practical wisdom to make moral decisions in life, which entails deliberating on the details of a given situation and deciding accordingly using deductive reasoning (Celano 2015). For Aristotle, a virtuous action must also be based on a virtuous purpose (Aristotle 2014). Virtuous purpose means undertaking an activity to honor the *telos* or the intrinsic ends of the domain to which that activity belongs, rather than to achieve any further aim (Bruni and Sugden 2013). For example, a virtuous person would practice medicine for its intrinsic aim to promote health, rather than an extrinsic motive such as earning money and status.

The relationship between virtue ethics and humanitarianism is most explicit in the principle of humanity. Humanity is both a value and a virtue. It expresses the fundamental value of a human life and a human person; at the same time, it expresses the virtue of being humane (Slim 2015a). Humanity as a virtue aligns with virtue ethics as it describes the moral character of the helper. In other words, humanitarian action is the expression of the virtue of being humane. According to philosopher Thomas Aquinas, humanity is related to a feeling of compassion, which arises when one is affected by other people’s suffering because of our shared condition of being human and moves one to alleviate another’s suffering (Ryan 2010). Humanity as a virtue also entails treating the recipients of aid with dignity and respect and from a place of equality and proximity (Fast 2015). Slim (2015a) commends virtue ethics as a more nuanced approach that allows for human emotions as compared to purely rational or calculative approaches such as deontological or consequentialist ethics. When making ethical decisions, Slim encourages humanitarians to find Aristotle’s mean and avoid extremes, while remaining true to the *telos* of humanitarianism – that is to alleviate the suffering of affected populations (Slim 2015a).

From a virtue‑ethics perspective, the ethical standing of innovative financing mechanisms depends on the motives and character of the actors involved. A key question is whether participants act for the intrinsic good of helping others rather than for external gains. This tension is especially pronounced for market actors, whose profit orientation may appear fundamentally misaligned with virtue‑ethical requirements. While internal motives are difficult to ascertain, virtue ethics allows assessment through observable conduct: whether these actors demonstrate intellectual and moral virtues in practice, exercise practical wisdom in balancing commercial and humanitarian aims, and adhere to Aristotle’s ‘golden mean’ by avoiding extremes such as excessive profit‑seeking or performative altruism.

#### Deontological ethics

Immanuel Kant, eighteenth century German philosopher, argued that some actions are morally right in and of themselves, regardless of their consequences or the moral character of the agent. Such inherently good actions are performed out of duty, which he contrasts with acting in adherence with duty (Kant 2002). For example, a person who is beneficent to others because he has a ‘sympathetic’ soul and enjoys an ‘inner gratification’ in helping others is acting ‘in conformity with duty,’ but there is actually nothing ‘morally meritorious’ in such a person because he is acting only for his own gratification (Guyer 2007, p. 45). Therefore, a morally worthy act is not dependent on one’s own circumstances but is unconditional and performed solely from duty. Central to Kantian ethics is a two-part principle called the categorical imperative. Its first formulation, the universal maxim, holds that a moral action must be universalizable through reasoning (Kant 2002, p. 18). For example, lying is categorically forbidden since one cannot rationally will a world in which everyone lies. The second formulation of the categorical imperative stipulates that we treat humanity ‘always at the same time as ends and never simply as means’ (Kant 2002, p. 47). Kant argued that all human beings have inherent dignity because of their ability to make free rational choices, and that we should never treat other humans merely as a means to our ends, thus undermining their dignity (Law 2007).

Another prominent deontological theorist is Scottish philosopher William David Ross. In contrast to Kant’s universalist approach, Ross puts forward a pluralistic approach by introducing seven morally positive duties: promise-keeping, reparation, gratitude, justice, beneficence, self-improvement, and non-maleficence (Ross 1930). These duties are known as *prima facie* or based on first impression – so called because they are binding at first glance but can come into conflict with each other. Ross does not provide a higher rule similar to Kant’s universal maxim to resolve such conflict between competing duties; instead, one must use discernment and judgment to determine which duty is most compelling in a given situation, reminiscent of Aristotle’s practical wisdom (Rawling 2023). Therefore, Ross offers a more flexible deontological approach that acknowledges complex realities, as opposed to Kant’s rigid approach that does not allow exceptions to the categorical imperative.

Deontology is closely intertwined with humanitarian ethics. While humanity as a virtue aligns with virtue ethics as explained above, humanity as a value is rooted in deontological ethics. Pictet used Kant’s second formulation of the categorical imperative to describe the principle of humanity: ‘Everyone shall be treated as a human being and not as an object, as an end in himself and not as a mere means to an end’ (Pictet 1979, p. 26). From this value of human life arises the duty to protect and preserve life, a concept known as the ‘humanitarian imperative.’ In other words, humanitarian action in the face of human suffering is obligatory because of our collective humanity (Rufini and Ozerdem 2005). Furthermore, deontological ethics have played a role in the evolution of humanitarianism. The adoption of a rights-based approach in humanitarianism from the 1950s shifted the moral emphasis from the charitable and compassionate ethic of ‘giving,’ i.e. the virtue of the helper, to the entitlement of affected populations (Gordon and Donini 2015; Slim 2015a).

When evaluating the ethics of market-based financing mechanisms, Kantian deontology would examine whether such instruments treat affected populations merely as a means to an end – such as vehicles for generating investor returns – rather than respecting their intrinsic worth. As with virtue ethics, the underlying motives of actors are difficult to ascertain; therefore, observable elements such as discourse and institutional practices offer valuable clues. For instance, if beneficiaries are primarily described or treated as data points for performance metrics, this may signal ethical concerns. Ross’s pluralistic deontology offers an additional ethical lens by emphasizing multiple duties. This framework prompts reflection on whether such mechanisms uphold the duty of beneficence by genuinely prioritizing the well-being of affected populations; respect the duty of non-maleficence by ensuring that vulnerable individuals are not excluded from or harmed by assistance; and honor the duty of justice by promoting fair risk-sharing, equitable distribution of benefits and burdens, and the prevention of exploitation.

#### Consequentialist ethics

Consequentialism is an umbrella term encompassing all ethical reasoning that judges an action to be morally right if it produces good results (O’Neill 2008). The most well-known and classical consequentialist theory is Jeremy Bentham’s utilitarianism. Bentham judged the morality of an action according to the principle of utility, or the tendency of an action to augment or diminish the happiness of the parties whose interests are in question (Bentham, 1996). Although Bentham was not the first philosopher to use the phrase ‘the greatest happiness of the greatest number,’ his contribution was in using the principle of utility as a moral standard by which to judge the merits of acts, laws, and institutions (Bentham, 1996). In particular, Bentham proposed a quantitative approach to evaluate the morality of an action by calculating the aggregate happiness generated, i.e. the total amount of pleasure created and pain reduced for all those affected by the action (Law 2007).

Recognizing that humanitarian aid can at times cause more harm than good, scholars have employed consequentialist reasoning to argue that good intentions alone are insufficient, urging agencies to assess the actual outcomes of their interventions (Anderson 1999; Terry 2002; Rubenstein 2015). Consequentialist ethics started to play a more prominent role in humanitarian practice following the failures of the humanitarian system to respond effectively to large-scale emergencies of the 1960s-80 s such as the Biafra-Nigerian war and the Ethiopian famine (Gordon and Donini 2015). In the aftermath of these system-wide failures, humanitarian actors shifted from deontological to consequentialist ethics to guide their practice, driven by a growing concern with the negative consequences of humanitarian action and increasing demand to measure outcomes and effectiveness (Barnett 2022).

Consequentialist ethics align with the motivations driving the adoption of innovative financing mechanisms, which seek to mobilize new resources, optimize aid delivery, accelerate the disbursement of funds, or achieve some other form of improvement over the status quo. Whereas deontological ethics would require aid agencies to uphold humanitarian principles, a consequentialist stance could judge a financing model that compromises those principles to be ethically defensible if it demonstrably produces the greatest overall good. However, classical utilitarianism’s egalitarian framework, which asserts that ‘everybody is to count for one and nobody as more than one’ (MacIntyre 2002, p. 226), creates ethical ambiguities when applied to innovative humanitarian finance. Under this approach, the morality of a financing mechanism would hinge on aggregating happiness across all stakeholders – affected populations, private sector partners, donors, and implementing organizations – without distinguishing between their roles or vulnerabilities. By treating all happiness as morally relevant and equivalent, classical utilitarianism gives equal weight to commercial interests and humanitarian needs, contradicting humanitarian principles as ‘there is no greater goal beyond the person in humanitarian action’ (Slim 2015a, p. 47).

Instead, the proposed analytical framework draws on Peter Singer’s preference utilitarianism to foreground the needs of affected populations. Unlike classical utilitarianism, which seeks to maximize overall happiness, preference utilitarianism prioritizes actions that best fulfill the preferences of those directly impacted (Singer 2011). This ethical stance necessitates meaningful consultation with affected communities to ensure that financing mechanisms are responsive to their expressed needs and preferences. While Singer holds that the preferences of those impacted merit equal consideration, he underscores the primacy of basic needs, such as food, shelter, and safety. Accordingly, from a preference utilitarian perspective, the imperative to meet these essential needs takes precedence over commercial or institutional interests. Grounding innovative humanitarian finance in the priorities of affected populations, rather than abstract utility calculations, thus offers a more ethically robust framework that addresses normative questions of justice overlooked by classical utilitarianism. Table 1 below summarizes the moral philosophical traditions discussed in this section.

**Table 1:** Moral philosophical traditions and their application to innovative humanitarian finance

Key philosophers	**Virtue ethics**	**Deontological ethics**		**Consequentialist ethics**	
	Aristotle	Immanuel Kant	William David Ross	Jeremy Bentham	Peter Singer
How is morality understood?	Expression of virtue	Obedience to duty		Utility to generate happiness	Utility to satisfy preferences
What constitutes a morally right action?	Actions that are in character for a virtuous person	Actions that are performed out of duty	Actions rooted in *prima facie* duties	Actions that lead to the greatest happiness for the greatest number	Actions that maximize the preferences of those affected
How would it appraise the ethics of innovative humanitarian finance?	Examine the character and motives of actors involved	Whether they are undertaken out of duty and treat humanity as an end, not as a means	Whether they adhere to *prima facie* duties	Whether they generate a net positive aggregate happiness for those affected	Whether they fulfill the preferences, especially basic needs, of those affected

## Presenting the analytical framework

Building on the ethical perspectives outlined above, this section introduces the analytical framework. The article proceeds from the premise that many innovative financing mechanisms effectively create new markets by transforming humanitarian action into investment or insurance products. Accordingly, market ethics provide the foundational point of departure. The framework comprises five parameters, developed in dialogue with Satz and Sandel’s theories, but adapted to the distinctive ethical tensions inherent in innovative humanitarian finance. Relevant humanitarian principles and moral philosophical approaches are subsequently layered onto this foundation.

The analytical framework is most appropriate for financing instruments that introduce private capital, return expectations, and market‑based incentive structures into humanitarian action. This includes impact bonds, catastrophe bonds, refugee investment funds, and insurance‑based risk‑transfer mechanisms, all of which embed profit motives and create the kinds of power asymmetries, incentive distortions, and potential misalignment with humanitarian priorities that the framework is designed to interrogate. By contrast, instruments that do not involve return‑seeking investors – such as Islamic social finance, micro‑levies, or pooled donor funds – raise fewer market‑driven ethical concerns, making them only partially suited to the framework.

Ethical concerns such as information asymmetries, donor influence, and constraints on operational independence are also present in traditional humanitarian funding arrangements. However, the aforementioned market‑based instruments introduce additional and qualitatively distinct ethical questions linked to the presence of return‑seeking investors, financial incentives tied to outcome metrics, and transfer of performance risk to humanitarian actors, which warrant a dedicated analysis. These features can reshape program design, create pressures to prioritize ‘bankable’ beneficiaries, and embed commercial logics into humanitarian decision-making. Moreover, while traditional grant funding typically involves a bilateral relationship between a donor and an implementing agency, instruments such as impact bonds bring together a large constellation of actors – investors, outcome funders, implementers, and intermediaries – whose interests must be aligned for a deal to proceed. This high‑stakes, multi‑stakeholder environment increases the likelihood that humanitarian agencies feel compelled to compromise in order to secure financing, thereby intensifying ethical challenges that already exist within the sector.

Against this backdrop, I present the framework below through the case of impact bonds, beginning with a brief overview of the instrument before turning to the five parameters and their practical application.

### Overview of impact bonds

Impact bonds are a form of outcome‑based financing that differ from conventional bonds, as they lack fixed repayment obligations and instead tie payment to the achievement of measurable outcomes (Liang and Mansberger 2014; Fraser et al. 2018). Unlike input‑based financing such as grants, which allocate funds based on planned activities, outcome‑based financing disburses funds after outcomes are verified. A key argument for outcome-based financing is that switching the conditions of payment from activities to outcomes shifts service provider behavior and improves social outcomes for target populations (Economy, Carter and Airoldi, 2022). The innovation of impact bonds lies in the involvement of investors, who provide upfront capital and receive returns contingent on results, thereby shifting financial risk away from service providers and funding agencies (Broccardo et al. 2020). Impact bonds thus convene three main parties – investors, outcome funders, and implementing organizations – supported by intermediaries, evaluators, and financial advisors as needed.

Impact bonds are also part of the broader impact investment movement, which seeks to generate measurable social or environmental benefits alongside financial returns (Global Impact Investing Network 2025). While most impact investment is channeled through private equity – direct investments in private companies through equity stakes that carry no guarantee of repayment – impact bonds invest in social programs, with an outcome funder guaranteeing repayment if pre-defined outcomes are achieved (Gruyter et al. 2020; Hand et al., 2024). They gained prominence after the 2008 financial crisis, when governments such as the United Kingdom under fiscal austerity adopted social impact bonds (SIBs) to finance preventative programs in prison recidivism, homelessness, and health – areas with a potential for long-term reduction of public expenditures (Liang and Mansberger 2014; Joy and Shields 2017). Development impact bonds later emerged in low‑ and middle‑income countries, where donor governments or philanthropic foundations act as outcome funders (Alenda-Demoutiez 2020). As of May 2025, 259 impact bonds have been contracted across 40 countries, mobilizing $524 million in capital, though this remains a fraction of the $1.6 trillion global impact investment market (Global Impact Investing Network 2025; Gustafsson-Wright and Painter 2025).

Recent humanitarian applications have adapted impact bonds to conflict and displacement contexts. The ICRC launched the five-year Humanitarian Impact Bond in 2017 to finance physical rehabilitation centers for people with mobility impairments in Nigeria, Mali, and the Democratic Republic of the Congo. This initiative mobilized CHF 18.6 million in investor capital, with outcome funders pledging CHF 26.1 million, including up to 7% annual interest depending on how efficiently the new centers operated (Lau 2021). Following the ICRC pilot, the Refugee Impact Bond – initiated by KOIS Invest and implemented by the Near East Foundation – mobilized USD $9.8 million from investors to support the livelihoods of Syrian refugees and host communities in Jordan through micro‑enterprise creation. Outcome funders committed USD $13.8 million, with investors eligible for a maximum 5.1% annual return if the program met predefined targets related to business survival rates and household consumption levels (IKEA Foundation et al. 2021).

### The framework in practice

This section outlines how the analytical framework can be applied to assess the ethical dimensions of impact bonds through five parameters: (1) humanitarian actors’ understanding of the financing mechanism, (2) agency of humanitarian actors involved, (3) the extent to which the mechanism aligns with the needs of affected populations, (4) its distribution of costs and benefits, and (5) its implications for the intrinsic value of humanitarian action and the moral character of aid workers. Table 2 at the end of this section summarizes the framework and visually represents how the parameters integrate market ethics, humanitarian principles, and moral philosophy, while providing guiding questions for practitioners to support decision-making.

**Table 2:** Analytical framework applied to the use of impact bonds in humanitarian action (to be included after Sect. " Parameter 5: intrinsic value of humanitarian action" and before conclusion)

	Parameters	Market Ethics	Humanitarian Principles	Moral Philosophical Approaches	Guiding questions
1	Understanding of humanitarian actors	Unethical if humanitarian actors involved in impact bonds have weak understanding and asymmetrical knowledge of impact bonds vis-à-vis other parties	If humanitarian actors are unable to make informed, impartial decisions due to insufficient information and knowledge asymmetry, impact bonds would compromise their independence and ability to act in the best interest of the people they serve	Virtue ethics: An action based on weak understanding and poor judgment would demonstrate lack of intellectual virtues and therefore would not be in character for a virtuous person	What are humanitarian actors’ motivations for pursuing impact bonds? How do they understand impact bonds? Do they demonstrate sufficient knowledge of why and how one would use an impact bond? Is there knowledge asymmetry? Are there barriers to acquiring knowledge and information?
2	Agency of humanitarian actors	Unethical if humanitarian actors accept impact bonds under any terms offered due to resource and power differentials among the parties involved	Independence would be compromised if humanitarian actors do not have sufficient leverage to design impact bonds and negotiate the terms of their contract	Consequentialist ethics: Weak agency could lead to harmful consequences such as humanitarian actors accepting unfavorable terms and becoming vulnerable to exploitation Deontological ethics: The duty of justice requires fair distribution of benefits and burdens. It would not be just if one party gets an unfair deal or is exploited by more powerful parties	How much say do humanitarian actors have in designing impact bonds and negotiating the terms of their contract? Have humanitarian actors accepted unfavorable terms and conditions?
		Unethical if investors influence or pressure humanitarian actors to achieve outcomes so they can secure a return on their investment	Independence would be compromised if investors influence program delivery and interfere in humanitarian actors’ operational decisions	Virtue ethics would deem impact bonds ethical if investors are of virtuous character, i.e. if they are intrinsically motivated to advance humanitarian aims, rather than extrinsically motivated to make profit	To what extent do humanitarian actors have the freedom to make operational decisions independently? Do investors attempt to steer program delivery?
3	Needs of affected populations	Unethical if impact bonds prioritize outcomes or contexts that easily generate a return for investors over the needs of affected populations	Humanity would be undermined if: (1) impact bonds prioritize investor-aligned outcomes and metrics rather than what really matters to affected populations; (2) impact bonds avoid high-risk contexts, excluding the most vulnerable crisis-affected populations	Deontological ethics: The duty of beneficence requires individuals to act in ways that better the conditions of others and promote the maximum aggregate good. Outcomes that prioritize investor returns over the conditions of affected populations would not be aligned with this duty	How are the outcomes and operational contexts selected? To what extent is the selection driven by needs as opposed to achievability and measurability of outcomes?
		Unethical if the incentive to achieve program targets and pay out investors leads to gaming or other shortcuts that undermine the needs of aid recipients	Impartiality would be undermined if beneficiaries are selected on the basis of criteria other than their needs, such as their abilities or likelihood of success	Consequentialist ethics: Gaming practices such as cherry-picking program participants would result in harmful consequences, namely the exclusion of vulnerable individuals that most need aid Deontological ethics: The duty of non-maleficence would require that vulnerable individuals and communities are not excluded from assistance	How are aid recipients selected? Are there any indications of gaming, such as cherry-picking or parking certain cases? What safeguards exist against such risks?
4	Cost–benefit analysis	Unethical if impact bonds transfer resources to the private sector without meaningful benefits for affected populations	Humanity would be undermined if impact bonds divert humanitarian funds to private sector actors without proportional benefits for aid recipients	Consequentialist ethics: Ethicality of impact bonds depends on whether their consequences are net positive or negative. Significant benefits, such as bringing in new funds or improving humanitarian outcomes, could morally justify costs. If impact bonds divert limited humanitarian resources to the private sector without proportional benefits for affected populations, they would result in systemic harm	How does the cost of impact bonds compare with similar programs financed through conventional instruments? Do impact bonds deliver better outcomes than what could have been achieved through conventional financing? How innovative or risky are the programs financed through impact bonds? Do impact bonds finance different outcomes that could not have been achieved through conventional instruments? Do impact bonds add any other value?
5	Intrinsic value of humanitarian action	Unethical if impact bonds corrode the intrinsic value of humanitarian action	Humanity as a value would be undermined if humanitarian action is pursued as a means to another end, not because protecting human life and dignity is a fundamental good in itself Neutrality would be compromised if humanitarian actors are seen as endorsing a specific political or economic ideology	Deontological ethics: Treating humanity as a means, not as an end in itself, would be deontologically unethical Virtue ethics: A humanitarian action that does not honor the *telos* or the intrinsic goal of humanitarianism, and instead undertaken for extrinsic reasons, would be unethical	Is humanitarian action financed through an impact bond intrinsically or extrinsically motivated? How do impact bonds affect the intrinsic value, character, and meaning of humanitarian action?
		Unethical if tying payment to the achievement of outcomes leads aid workers to become fixated on quantifiable targets at the expense of unquantifiable dimensions of aid	Humanity would be undermined if aid workers solely focus on performance targets and do not treat aid recipients with respect, dignity, and compassion	Deontological ethics: Reducing aid recipients to metrics and targets, rather than treating them as moral subjects, would be unethical Virtue ethics: Impact bonds would be unethical if they undermine the moral character and virtues expected of aid workers	How do aid workers feel about impact bonds? Do impact bonds change how aid workers deliver aid to affected populations?

#### Parameter 1: understanding of humanitarian actors

The first parameter assesses the understanding of humanitarian actors about impact bonds. This parameter is derived from Satz’s (2010) argument that some parties in noxious markets have very weak or highly asymmetric knowledge about the goods being exchanged. If humanitarian actors involved in or pursuing impact bonds have weak understanding and poor information about this financing mechanism, and especially if knowledge asymmetry is present vis-à-vis other parties that puts humanitarian actors at a disadvantage, impact bonds would meet one of the criteria of an unethical market. Moreover, limited understanding risks undermining the humanitarian principle of independence. If humanitarian actors are unable to make informed, impartial decisions due to knowledge asymmetry and insufficient information, impact bonds would compromise their independence and ability to act in the best interest of the people they serve.

From a virtue ethics perspective, an action based on weak understanding and poor judgment would demonstrate lack of intellectual virtues and thus would not be in character for a virtuous person. However, Aristotle makes a distinction between ‘acting owing to ignorance’ and ‘acting in ignorance’ (Aristotle 2014). In the former, mistaken understanding or insufficient knowledge leads an agent to act differently than they would if fully informed, and such actions are deemed involuntary and not blameworthy. In the latter, diminished awareness prevents the agent from grasping the circumstances of their action, and this self‑induced ignorance is inexcusable since the agent was otherwise capable of informed choice. Therefore, a virtue ethicist would consider whether humanitarian actors’ limited understanding of impact bonds arose from circumstances beyond their control, or whether they had the capacity to grasp the situation but failed to do so – for instance, by neglecting to conduct adequate research and develop a robust understanding of impact bonds before adoption.

#### Parameter 2: agency of humanitarian actors

The second parameter assesses the agency of humanitarian actors involved in impact bonds. This parameter stems from Satz’s (2010) argument that the participants in noxious markets have substantially different resources and capacities, and that the weaker party is likely to agree to any terms offered, is vulnerable to exploitation, or cannot act independently. Specifically, the framework assesses the agency of humanitarian actors at both design and implementation stages, examining their ability to negotiate the terms of exchange as well as their ability to implement impact bonds independently without interference from other parties. If humanitarian actors do not have sufficient leverage in designing impact bonds and negotiating the terms of their contract due to resource and power differentials among the parties involved, or if investors pressure humanitarian actors to achieve program outcomes so they can secure a return on their investment, impact bonds would be an unethical market arising from the weak agency of humanitarian actors.

The SIB literature indicates that investors can influence program design as they often have a say in choosing service providers and in setting program outcomes and targets (Joy and Shields 2018; Gallucci et al. 2022). Additionally, impact bond investors safeguard their interests through strategies such as disbursing funds to service providers in performance-linked tranches and embedding contract termination rights in case the program deviates from agreed-upon outcomes (Burand 2019). Therefore, investors can, in principle, influence service providers’ behavior through contractual terms in a manner that puts pressure on them to achieve program targets. Therefore, the framework explores whether investors in impact bonds engage in such attempts to influence the design and delivery of humanitarian actions.

If investors interfere in program design and operational decisions – such as defining priorities, selecting beneficiaries, and determining delivery methods – this would undermine the humanitarian principle of independence. When formulating the principle of independence, Pictet aimed to shield humanitarianism from the ‘intrusion of politics’ (Pictet 1979, p. 62). However, if innovative financing mechanisms permit market actors to influence humanitarian decisions and operations, this raises concerns about the ‘intrusion of markets.’

This parameter spans all three moral philosophical approaches. From a virtue ethical lens, investors’ behavior during program design and implementation would reveal their character and motives – whether they are driven by a genuine commitment to humanitarian aims rather than by profit, and whether they exhibit virtues such as non-interference in aid delivery and respect for the independence of humanitarian actors. From a deontological standpoint, this parameter aligns with Ross’s duty of justice, which mandates that benefits and burdens be distributed fairly. An impact bond that creates an unfair advantage for some parties allowing for the exploitation of the weaker party would violate the duty of justice. Lastly, consequentialist ethics would complement these questions of character and fairness by examining the outcomes. If weak agency results in harmful effects – such as humanitarian actors accepting unfavorable terms, becoming vulnerable to exploitation, and ultimately failing to serve affected populations effectively – impact bonds would be deemed unethical from a consequentialist perspective.

#### Parameter 3: needs of affected populations

The third parameter examines the implications of impact bonds on the needs of affected populations. This parameter draws from Satz’s (2010) argument that some noxious markets produce harmful outcomes for individuals, leaving the affected individuals destitute or undermining their basic needs and interests. Impact bonds could potentially undermine the needs of affected populations in two ways: (1) Private sector involvement could result in prioritization of outcomes or contexts that easily generate a return for investors over the needs of affected populations; and (2) The incentive to achieve program outcomes and pay out investors could lead to gaming or other shortcuts that exclude those who most need assistance.

If impact bonds focus on investor-aligned outcomes, particularly outcomes that are easier to achieve and measure, rather than outcomes that would bring about the most meaningful change in people’s lives, or if they avoid high-risk contexts where likelihood of success is low, impact bonds would undermine the principle of humanity. Diverting resources from the needs articulated by affected populations toward outcomes that are more likely to serve the commercial interests of investors would also violate accountability to affected populations, embodied in the principle of humanity. Moreover, the principle of impartiality would be undermined if aid recipients are selected on the basis of criteria other than needs, such as their abilities or likelihood of achieving a certain outcome.

Indeed, the SIB literature indicates that implementers can engage in ‘gaming’ practices to achieve program outcomes more easily, such as ‘cherry-picking’ participants who are most likely to succeed, ‘parking’ participants deemed less likely to succeed thus giving them less priority or minimum possible services, or choosing ‘in-betweeners’ – those who have needs but can still be ‘saved’ (Andreu 2018; Joy and Shields 2018; Carter 2021; Fraser, Knoll and Hevenstone, 2022). Service providers may resort to gaming practices under investor pressure or to mitigate their own reputational and financial risks, particularly when they are liable for part of the loss. Given these risk factors, the framework would analyze whether financial incentives in impact bonds affect how humanitarian actors select aid recipients.

Within deontological ethics, Ross’s (1930) prima facie duties is relevant to this parameter. The duty of beneficence requires agents to act in ways that better the conditions of others and promote the maximum aggregate good. Outcomes that prioritize investor returns over the conditions of affected populations would not be aligned with the duty of beneficence. Additionally, gaming practices such as cherry-picking could result in harmful consequences, namely the exclusion of vulnerable individuals and households from assistance. This would be unethical according to both consequentialist ethics as well as the Rossian duty of non-maleficence or the duty to avoid causing harm to others.

#### Parameter 4: cost–benefit analysis

The fourth parameter includes a cost–benefit analysis to examine whether the additional costs of financing a humanitarian action through an impact bond bring proportional benefits. This parameter is linked to Satz’s (2010) argument that noxious markets can produce harmful outcomes not only for the affected individuals, but also for society at large by undermining social norms and values. Impact bonds would be harmful for society if existing humanitarian resources, which could have been used to assist people in need, are transferred to the private sector through investor returns and legal and administrative fees without meaningful benefits. The significantly higher costs of impact bonds would be justified only if they deliver better outcomes than traditional financing models or outcomes that are otherwise unachievable.

The principle of humanity, rooted in the commitment to protect life and health and alleviate suffering, implies that every available resource should be channeled directly toward helping those in need as much as possible. If substantial new costs are incurred to set up a complex financing mechanism and cover investor premiums, fewer resources would reach affected populations, which would be especially problematic if these costs do not translate into demonstrably better outcomes than those achievable through traditional financing models. Such resource allocation would contradict the principle of humanity by prioritizing financial returns over optimizing aid for affected populations.

This parameter is fundamentally linked to consequentialist ethics as it weighs the costs and benefits of impact bonds to understand if their consequences are net positive or negative. If impact bonds bring considerable benefits such as increasing available funds or improving humanitarian outcomes, their higher costs could be morally justified from a consequentialist perspective. On the contrary, if impact bonds divert humanitarian funds without adding tangible value, it would result in a systematic harm, thus failing the consequentialist ethical test. Although it is possible that impact bonds bring benefits for other actors – such as improving aid transparency for donors or enhancing the performance and internal systems of humanitarian agencies, this parameter is primarily concerned with benefits for affected populations in line with preference utilitarianism.

#### Parameter 5: intrinsic value of humanitarian action

This parameter examines whether financing a humanitarian action through impact bonds potentially corrodes its intrinsic value, drawing from Sandel’s (2012) theory that commodifying some goods can corrupt their value and alter their meaning. Sandel argues that such commodification can change people’s attitudes toward the goods being exchanged and crowd out moral values and norms. Accordingly, this parameter investigates the following: (1) whether impact bonds corrode the intrinsic value and alter the character of humanitarian action, and (2) whether tying payment to the achievement of measurable outcomes could lead aid workers to become fixated on quantifiable targets at the expense of unquantifiable, relational dimensions of aid.

As discussed earlier, the principle of humanity embodies the intrinsic value of human life and dignity and stipulates that protecting this value is the ultimate goal of humanitarian action. If humanitarian action is pursued not for its intrinsic purpose but as a means to another end, such as facilitating private investment in fragile contexts, the principle of humanity risks being compromised. Additionally, aid delivery that solely focuses on efficiency and quantifiable targets and de-prioritizes unquantifiable virtues such as compassion for and solidarity with aid recipients would undermine the principle of humanity.

This parameter also carries implications for the principle of neutrality, which requires that humanitarian actors refrain from engaging in ideological controversies. The origins of SIBs were embedded in a wider neoliberal political project that promoted the shrinking of the state and privatization of social services, on the assumption that markets are more effective and efficient (Joy and Shields 2017). Impact bonds are therefore rooted in neoliberalism and free-market capitalism – ideologies that are not universally accepted. Consequently, if communities perceive humanitarian aid as part of a profit-driven model or as an endorsement of specific political or economic ideologies, their trust in humanitarian actors may erode. Therefore, Sandel’s theory that commodification alters people’s attitudes toward the goods being exchanged is applicable to aid recipients, as much as aid workers.

This parameter engages with both deontological and virtue ethics. If impact bonds change the attitudes of aid workers, leading them to treat aid recipients as mere metrics and targets rather than as moral subjects, such a shift would violate Kant’s categorical imperative, which demands that humanity always be treated as an end in itself, never merely as a means. Virtue ethics would also deem this shift in attitudes unethical, as it would signal a decline in the moral virtues expected of aid workers. Additionally, a humanitarian action that fails to honor the *telos* or the intrinsic goal of humanitarianism, and is instead pursued for extrinsic motivations, would not fulfill Aristotle’s concept of virtuous purpose.

## Conclusion

This article seeks to advance knowledge about innovative humanitarian finance by proposing an analytical framework for examining the ethical dimensions of new financing models in humanitarian action. By adopting an ethical lens, the article moves beyond questions of technical and financial performance to ask how innovative financing mechanisms work in humanitarian settings, for whom they work, and at what cost. The proposed framework offers breadth by bringing together interdisciplinary insights from market ethics, humanitarian ethics, and moral philosophical traditions, and depth by examining the underlying conditions of financing mechanisms such as power and resource dynamics, their effects on aid recipients, and the ways in which such humanitarian-business partnerships reshape the nature and meaning of humanitarianism. While the application of the framework is demonstrated through the case of impact bonds, it can be adapted for an ethical appraisal of other blended-financing models that involve return-seeking private sector partners.

A limitation of the framework is that its conceptual components do not always align seamlessly. For instance, a financing instrument may not meet the criteria of an unethical market yet still compromise one or more humanitarian principles. As a result, applying the framework does not yield a single, definitive judgement about the ethicality of a given mechanism. Rather, different ethical benchmarks may point in different directions, each highlighting distinct implications and trade‑offs. Despite these tensions, the framework offers a valuable integrative lens: by drawing on diverse ethical traditions, it avoids reducing complex financing arrangements to a single evaluative standard and instead ensures that innovative mechanisms are examined from all relevant angles. Additionally, the framework helps distinguish between ethical challenges that stem from the inherent features of a financing model and those that arise from specific design or implementation choices. When the latter is the case, the implication is that such mechanisms need not be rejected outright; instead, ethical risks may be mitigated through appropriate safeguards.

## Data Availability

No datasets were generated or analysed during the current study.
